# Late Central Nervous System Relapse in a Patient with Maxillary Sinus Lymphoma

**DOI:** 10.7759/cureus.3745

**Published:** 2018-12-18

**Authors:** Tariq Kewan, Hassan Awada, Fahrettin Covut, Abdo Haddad, Hamed Daw

**Affiliations:** 1 Internal Medicine, Cleveland Clinic - Fairview Hospital, Cleveland, USA; 2 Hematology and Oncology, Cleveland Clinic - Fairview Hospital, Cleveland, USA

**Keywords:** diffuse large b cell lymphoma, cns relapse, maxillary sinus lymphoma

## Abstract

Paranasal sinus lymphoma (PNL) is a rare presentation of extranodal non-Hodgkin lymphoma (NHL) with a natural history different from other types of lymphoma. The maxillary sinus is the most common paranasal sinus involved in NHL. Involvement of the central nervous system (CNS) is a rare complication of PNL. In this case report, we present a case of diffuse large B cell lymphoma (DLBL) that developed in the left maxillary sinus and relapsed as a left frontal brain mass after four years of disease remission.

## Introduction

Head and neck are the second most common extranodal sites involved in non-Hodgkin lymphoma (NHL). Paranasal sinus lymphoma (PNL) is a rare presentation of extranodal NHL with a natural history different from other types of lymphoma. The incidence of NHL arising from the sinonasal tract ranges from 0.2% to 2% of all lymphoma cases in the western countries. The maxillary sinus is the most common paranasal sinus involved in NHL followed by the ethmoid, sphenoid, and the frontal sinuses, respectively. The most common PNL sub-type is diffuse large B cell lymphoma (DLBL), accounting for two-thirds of the cases. The involvement of the central nervous system (CNS) is a devastating and rare complication of PNL. In this report, we present a case of DLBL that developed in the left maxillary sinus and relapsed as a left frontal brain mass after four years of disease remission. 

## Case presentation

**Figure 1:**
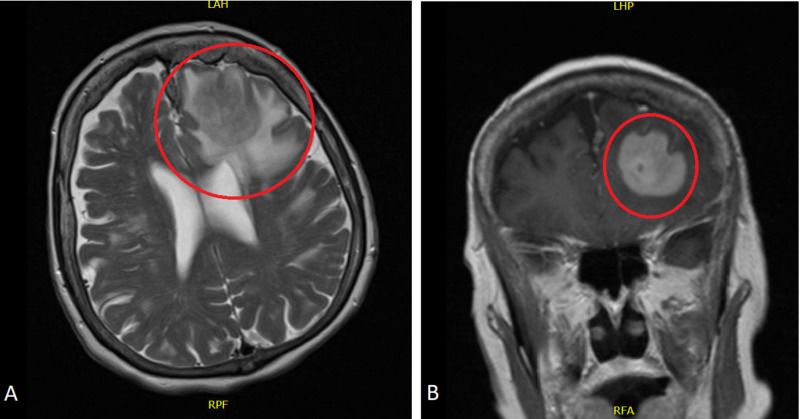
Magnetic resonance imaging of brain with contrast Horizontal (A) and coronal (B) sections of brain MRI showing a dominant left frontal mass (red circles) approximately 3.8 cm in diameter with adjacent rim of vasogenic edema MRI: magnetic resonance imaging

A 75-year-old female patient was admitted to the emergency department with a sudden-onset tonic-clonic seizure and status epilepticus in December 2017. She had a history of left maxillary sinus DLBL diagnosed in July 2009. The patient was intubated and admitted to the medical intensive care unit (MICU) for the management of status epilepticus. Magnetic resonance imaging (MRI) of the brain with contrast revealed a dominant left frontal mass approximately 3.8 cm in diameter with an adjacent rim of vasogenic edema (Figure 1). Signal characteristics, restricted diffusion, and the pattern of enhancement raise question of lymphoma, metastasis, or less likely intermediate-grade primary brain tumor, given the multifocal disease.

In July 2009, she was admitted with seven weeks of left facial swelling, erythema, and pressure sensation. 18F-fluorodeoxyglucose (FDG) positron emission tomography/computed tomography (PET/CT) scan demonstrated 2.3 x 1.4 cm focus anterior to the left maxilla. A biopsy was done by oral surgery, and the definitive pathology diagnosis was DLBL with positive immunohistochemical stain for CD20, BCL2, LCA, and CD45RB. The bone marrow biopsy was negative. The patient was staged as stage IIA with a CNS international prognostic index (CNS-IPI) score of four (high-risk group). Three cycles of chemotherapy with rituximab, cyclophosphamide, doxorubicin, vincristine, and prednisone (R-CHOP) regimen and 36 Gy involved-field radiation therapy were given. The post-chemo/radiotherapy PET/CT scan showed decreased size and activity of the subcutaneous soft tissue mass anterior to the left maxilla with a residual mass of 2.4 x 0.6 cm. This mass resolved on further follow-up.

Subsequent biopsy of the frontal lobe mass with frameless CT-guided navigation confirmed DLBL with positive immunohistochemical stain for CD10, CD20, BCL2, and MUM1. Over 80% of the cells stained for Ki-67 and 70% were positive for MYC. They were negative for CD3 and BCL6. The immunophenotype of the neoplastic cells was not specific for primary CNS large B-cell lymphoma, raising suspicion for CNS involvement by lymphoma originating at a different location. The patient received five cycles of high-dose methotrexate and rituximab. Two weeks after the last cycle, she had status epilepticus again and was admitted to the MICU and intubated. Brain MRI with contrast showed right frontal and parietal infarcts with a significant progression of frontal lymphoma. Chemotherapy was stopped, and the patient was started on temozolomide 150 mg/m^2^ since May/2018.

## Discussion

Head and neck are the second most common extranodal sites involved in NHL. PNL is a rare presentation of extranodal NHL with a natural history different from other lymphoma types. The incidence of NHL arising from the sinonasal tract ranges from 0.2% to 2% of all lymphoma cases in the western countries. The maxillary sinus is the most common paranasal sinus involved in NHL followed by the ethmoid, sphenoid, and the frontal sinuses respectively. The most common sub-type is DLBL, accounting for two-thirds of the cases. CNS involvement is a devastating and rare complication of PNL [1-2]. 

Short case series and case reports of PNL have been reported, although the paranasal sinuses are rare primary sites for NHL. In most of the published reports, the disease presents with local signs and symptoms and is limited to stage IE or IIE. In a series of 14 patients with PNL, all patients were at stage I-II. The most common type was DLBL in six cases. The primary involvement sites included the maxillary sinus (11 cases), the ethmoid sinus (two cases), and the sphenoid sinus (one case) [3]. In another report of 14 cases, all patients were at stage I-II [4]. In another case report of maxillary sinus lymphoma, the patient presented with non-tender facial swelling and an intermittent mild toothache [5].

Relapses of PNL involve the primary affected site, CNS, or both. CNS relapse occurs in cases where the invading lesion disturbs the local barrier and exposes the meninges and the CNS to the invading nearby lesion [6]. In one case series, seven out of 14 patients with PNL relapsed in the follow-up period; four of the relapses involved the CNS. A few studies presenting PNL and CNS relapse have been summarized in Table 1. In a case report of a 58-year-old female patient with right maxillary sinus lymphoma, the patient had CNS relapse with a left pontine lesion that was removed and found to be consistent with DLBL. A few days later, she suffered from right limb weakness and was found to have cervical spinal cord metastasis [4-5].

**Table 1:** Incidence of central nervous system relapse in patients with paranasal sinus lymphoma in four studies R-CHOP: rituximab, cyclophosphamide, doxorubicin, vincristine, and prednisone; CNS: central nervous system

Study	Total number of patients	Treatment options	Intrathecal chemotherapy	Patients with CNS relapse
Oprea C et al. [4]	14	CHOP	No one received CNS prophylaxis	4
Laskin JJ et al. [6]	44	CHOP and radiotherapy	Methotrexate/cytarabine in 26 patients only	3
Lee GW et al. [7]	80	R-CHOP, R-CHOP/Radiotherapy	Methotrexate (In nine patients), methotrexate/cytarabine/steroid (in three patients)	1
Proulx GM et al. [8]	23	Radiotherapy, CHOP /Radiotherapy	No one received CNS prophylaxis	2

CNS-IPI is a prognostic model used to assess the risk of CNS disease (Table 2). The CNS-IPI is a highly reproducible tool that can be used to estimate the risk of CNS relapse or progression in patients with DLBL treated with R-CHOP chemotherapy. Approximately 90% of the patients with DLBL belong to the low- and intermediate-risk groups and have a CNS relapse risk of less than 5%. By contrast, patients in the high-risk group have more than 10% risk of CNS relapse and should be considered for CNS-directed investigations and prophylactic interventions [9-10]. Our patient has a CNS-IPI of four and fits within the high-risk group of patients.

**Table 2:** Central nervous system international prognostic index CNS-IPI is a reproducible tool that can be used to estimate the risk of CNS relapse or progression in patients with DLBL. DLBL: diffuse large B cell lymphoma, CNS-IPI: central nervous system international prognostic index, CNS: central nervous system

Prognostic model to assess the risk of CNS disease	Points
Age > 60 years	1
Serum LDH > normal	1
Performance Status >1	1
Stage III or IV	1
Extranodal involvement >1	1
Kidney or adrenal gland involvement	1
Risk	Score
Low risk	0-1
Intermediate risk	2-3
High risk	4-6

The treatment protocol for PNL consists of a combination chemotherapy followed by involved-field irradiation in patients with good performance status. In a case series of 14 patients with PNL, two patients underwent total maxillectomy and 12 underwent local excision or biopsy. All patients received chemotherapy, and six patients received radiotherapy after chemotherapy. Both five-year and event-free survival rates were 78.6 %, with a median survival of 59.5 months. In another case series, 14 patients were treated with CHOP or CHOP-like chemotherapy regimen. Ten patients achieved complete remission and three achieved partial remission. With a median follow-up period of 80 months, six patients had died and eight patients remained alive [3-4].

It seems that R-CHOP chemotherapy regimen does not protect from CNS relapses in patients with NHL. Common strategies for CNS prophylaxis include intrathecal (IT) chemotherapy and systemic CNS penetrants such as methotrexate. IT chemotherapy does not adequately penetrate the brain parenchyma, and hence it is insufficient in preventing parenchymal CNS recurrences. Most experts recommended  systemic  methotrexate  for high-risk groups, which penetrates both the leptomeningeal and parenchymal CNS compartments. Even though systemic CNS prophylaxis is widely preferred over IT alone, its efficacy is unclear. Ongoing efforts in the search for appropriate CNS prophylaxis strategies are warranted. High-dose methotrexate in conjunction with R-CHOP chemotherapy can be used for eligible patients deemed at a high risk of CNS recurrence, especially those with high-risk CNS-IPI and extranodal involvement [11]. In our case, the patient was treated with three cycles of R-CHOP followed by involved-field irradiation therapy. We support the use of the CNS-IPI when deciding whether CNS prophylaxis is indicated or not.

## Conclusions

CNS involvement is a devastating and rare complication of PNL. The CNS-IPI is a highly reproducible tool that can be used to estimate the risk of CNS relapse or progression in patients with DLBL treated with R-CHOP chemotherapy. Systemic CNS chemoprophylaxis is widely preferred over IT chemoprophylaxis alone; however, its efficacy is unclear. Research concerning appropriate CNS prophylaxis strategies is warranted.

## References

[1] Fellbaum C, Hansmann ML, Lennert K (1989). Malignant lymphomas of the nasal cavity and paranasal sinuses. Virchows Arch A Pathol Anat Histopathol.

[2] Abbondanzo SL, Wenig BM (1995). Non-hodgkin's lymphoma of the sinonasal tract a clinicopathologic and immunophenotypic study of 120 cases. Cancer.

[3] Su ZY, Zhang DS, Zhu MQ, Shi YX, Jiang WQ (2007). Primary non-hodgkin's lymphoma of the paranasal sinuses: a report of 14 cases. Ai Zheng.

[4] Oprea C, Cainap C, Azoulay R (2005). Primary diffuse large B-cell non-hodgkin lymphoma of the paranasal sinuses: a report of 14 cases. Br J Haematol.

[5] Liu B, Dong L, Shi W, Lv J, Guo L, Liu M (2018). Primary maxillary sinus lymphoma. QJM.

[6] Laskin JJ, Savage KJ, Voss N, Gascoyne RD, Connors JM (2005). Primary paranasal sinus lymphoma: natural history and improved outcome with central nervous system chemoprophylaxis. Leuk Lymphoma.

[7] Lee GW, Go SI, Kim SH (2015). Clinical outcome and prognosis of patients with primary sinonasal tract diffuse large B-cell lymphoma treated with rituximab-cyclophosphamide, doxorubicin, vincristine and prednisone chemotherapy: a study by the consortium for Improving survival of lymphoma. Leuk Lymphoma.

[8] Proulx GM, Caudra-Garcia I, Ferry J (2003). Lymphoma of the nasal cavity and paranasal sinuses: treatment and outcome of early-stage disease. Am J Clin Oncol.

[9] Kansara R, Villa D, Gerrie AS (2017). Site of central nervous system (CNS) relapse in patients with diffuse large B-cell lymphoma (DLBL) by the CNS-IPI risk model. Br J Haematol.

[10] Schmitz N, Zeynalova S, Nickelsen M (2016). CNS international prognostic index: a risk model for CNS relapse in patients with diffuse large B-cell lymphoma treated with R-CHOP. J Clin Oncol.

[11] Kansara R (2018). Central nervous system prophylaxis strategies in diffuse large B cell lymphoma. Curr Treat Options Oncol.

